# MMP1 and MMP11 Expression in Peripheral Blood Mononuclear Cells upon Their Interaction with Breast Cancer Cells and Fibroblasts

**DOI:** 10.3390/ijms22010371

**Published:** 2020-12-31

**Authors:** Noemi Eiro, Sandra Cid, Nuria Aguado, María Fraile, Nagore de Pablo, Berta Fernández, Francisco Domínguez, Luis O. González, Francisco J. Vizoso

**Affiliations:** 1 Research Unit, Fundación Hospital de Jove, 33290 Gijón, Spain; sandra.cid.89@gmail.com (S.C.); maria.fraile82@gmail.com (M.F.); nagoredep@gmail.com (N.d.P.); 2 Department of Surgery, Hospital Universitario San Agustín, 33401 Avilés, Spain; n.aguadosuarez@gmail.com; 3 Department of Surgery, Hospital Universitario Central de Asturias, 33011 Oviedo, Spain; mbertafergo@gmail.com; 4 Department of Anatomical Pathology, Hospital Universitario de Cabueñes, 33394 Gijón, Spain; fdoig59@yahoo.es; 5 Department of Anatomical Pathology, Fundación Hospital de Jove, 33209 Gijón, Spain; a.patologica2@hospitaldejove.com; 6 Department of Surgery, Fundación Hospital de Jove, 33290 Gijón, Spain

**Keywords:** inflammation and cancer, inflammatory cells, stroma, breast cancer, PBMC

## Abstract

Tumor-infiltrating immune cells phenotype is associated with tumor progression. However, little is known about the phenotype of the peripheral blood mononuclear cells (PBMC) from breast cancer patients. We investigated MMP1 and MMP11 expression in PBMC from breast cancer patients and we analyzed gene expression changes upon their interaction with cancer cells and cancer-associated fibroblasts (CAF). We measured the impact of PBMC on proinflammatory gene expression in breast cancer cells, normal fibroblast (NF), and CAF and the impact on proliferation and invasiveness capacity of breast cancer cells. Gene expression of MMP1 and MMP11 in PBMC from breast cancer patients (*n* = 54) and control (*n* = 28); expression of IL1A, IL6, IL17, IFNβ, and NFĸB in breast cancer cell lines (MCF-7 and MDA-MB-231); and, additionally, IL10 and MMP11 in CAF and NF were analyzed by qRT-PCR before and after co-culture. Our results show the existence of a subpopulation of breast cancer patients (25.9%) with very high levels of MMP11 gene expression in PBMC. Also, gene expression of MMP1 and MMP11 increases in PBMC after co-culture with breast cancer cell lines, NF or CAF. PBMC from healthy or breast cancer patients induce an increased proliferation rate on MCF-7 and an increased invasiveness capacity of MDA-MB-231. Finally, we show a differential expression profile of inflammatory genes in NF and CAF when co-cultured with control or breast cancer PBMC. We have observed that MMPs’ expression in PBMC is regulated by the microenvironment, while the expression of inflammatory genes in NF or CAF is differentially regulated by PBMC. These findings confirm the importance of the crosstalk between stromal cells and suggest that PBMC would play a role in promoting aggressive tumor behavior.

## 1. Introduction

Breast cancer is a complex and heterogeneous disease. Neoformed tumor mass is constituted by cancer cells and by an important stromal component. Cancer-associated fibroblasts (CAF) and tumor-infiltrating immune cells are key cellular components of the tumor stroma. The latter derive from peripheral blood mononuclear cells (PBMC). The immune system plays paradoxical roles in tumorigenesis [1]. Historically, tumor-infiltrating immune cells were considered manifestations of an intrinsic defense mechanism against tumors [2]; however, it was evidenced that leukocyte infiltration can promote tumor progression by stimulating angiogenesis, cell proliferation, and invasiveness [3,4]. Matrix metalloproteases (MMPs) are zinc-dependent endopeptidases playing an important role in the degradation of the stromal connective tissue and basement membrane components, which are key elements during tumor invasion and metastasis. However, MMPs can participate in metastasis not only by remodeling the extracellular matrix, but also through their ability to cleave and activate growth factors, cell adhesion molecules, and cell surface receptor, resulting in an anti-apoptotic and/or pro-angiogenic effects [5].

In previous studies, we have proved that tumor-infiltrating immune cells expressing high levels of MMPs had a higher rate of distant metastasis development compared with tumors with low expression profile of MMPs in immune cells [6,7,8,9,10,11]. In particular, the expression of MMP11 in tumor-infiltrating immune cells was highly associated with both distant metastasis development and high inflammatory profile in breast cancer [10,11,12,13,14,15]. In addition, we have shown that CAF from MMP11 positive tumor-infiltrating immune cells tumors may overexpress tumor progression factors and may show higher breast cancer cell invasion and angiogenesis [16,17]. In line with this, MMP1 expression in immune cells at the sentinel lymph nodes was associated with sequential metastasis across lymph nodes in breast cancer [18]. All these data suggest that degradation capacity of MMP1 and other metalloproteases, which cleave several components of the extracellular matrix, could contribute to promoting tumor spread via the lymph nodes. 

Evidence suggests a contribution of tumor-infiltrating immune cells, depending on their phenotype, in the tumor behavior. However, little is known about the implication of PBMC phenotype in breast cancer, despite MMPs’ expression in different immune cells. Furthermore, PBMC would change their MMPs’ expression upon contacting the tumor microenvironment, generating different potential scenarios. For these reasons it is critical to determine whether the interaction between inflammatory cells with tumor cells or CAF may regulate MMPs expression.

In the present work, we have investigated the gene expression of MMP1 and MMP11 in PBMC from breast cancer patients (BC-PBMC), before and after co-culture with breast cancer cell lines or CAF, compared to PBMC from healthy women (C-PBMC). In addition, we have investigated the influence of BC-PBMC on the inflammatory profile of breast cancer cell lines, NF and CAF. Our results suggest that expression of MMPs in PBMC can be regulated by cancer cells and CAF, which may favor tumor progression. These findings confirm the importance of the interaction and communication between stromal cells in promoting aggressive tumor behavior.

## 2. Results

### 2.1. MMP1 and MMP11 Gene Expression in PBMC from Breast Cancer Patients and Healthy Women

**Figure 1:**
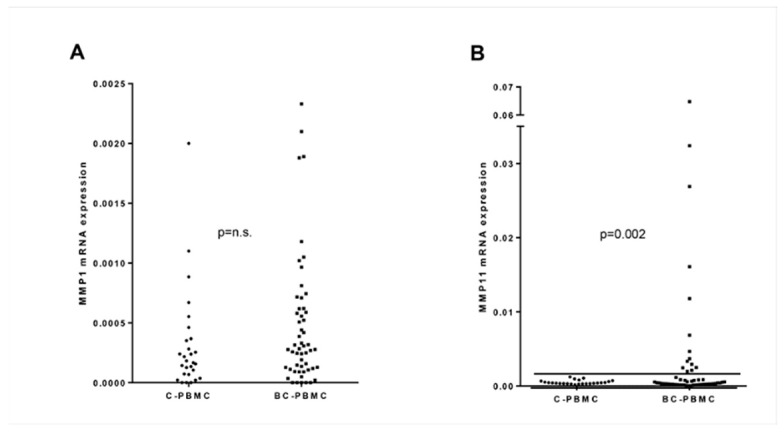
MMP1 and MMP11 gene expression in peripheral blood mononuclear cells (PBMC) from breast cancer patients. MMP1 (**A**) and MMP11 (**B**) gene expression in PBMC from breast cancer patients (BC-PBMC, *n* = 54) and healthy women (controls, C-PBMC, *n* = 28). The horizontal line marks an arbitrary threshold, corresponding to the highest MMP11 gene expression in C-PBMC, used as cut-off point. Significant difference was found between MMP11 gene expression in BC-PBMC from patients above the threshold and C-PBMC. (n.s.: not significant). Results of the qRT-PCR data were represented as ΔΔCT values.

Basal gene expression of MMP1 and MMP11 was studied in PBMC from breast cancer patients (BC-PBMC) and healthy women (controls, C-PBMC). No significant differences were observed between groups (Figure 1A,B). However, a closer observation of MMP11 gene expression showed higher dispersion in the BC-PBMC group. Then, we decided to establish an arbitrary threshold considering the highest MMP11 gene expression in C-PBMC as a cut-off point. This threshold allowed the identification of a subgroup of breast cancer patients (*n* = 14, 25.9% of total BC-patients) with significantly higher MMP11 gene expression (Figure 1B). This finding suggested that MMP11 gene expression in PBMC might contribute to identifying a subset of patients with breast cancer. However, no significant relationship was found between high levels of MMP11 gene expression in BC-PBMC with clinico-pathological characteristics defined in Table 3 (data not shown). This could indicate that MMP11 expression may depend on the response of the patient and not on the characteristics of the tumor.

### 2.2. MMP1 and MMP11 Gene Expression in PBMC after Co-Culture with Breast Cancer Cell Lines

**Figure 2:**
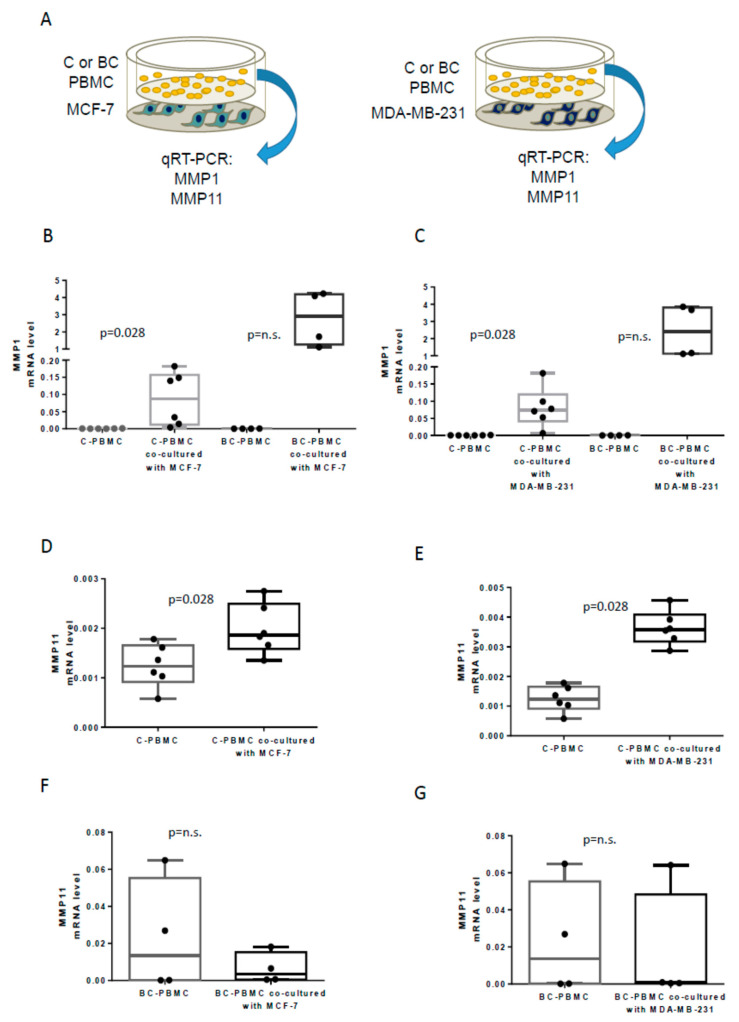
MMP1 and MMP11 gene expression in PBMC after co-culture with breast cancer cell lines. Scheme of experimental design (**A**). MMP1 gene expression in PBMC from healthy women (controls, C-PBMC, *n* = 6) and in PBMC from breast cancer patients (BC-PBMC, *n* = 4) before and after co-culture with MCF-7 (**B**) and MDA-MB-231 (**C**) breast cancer cell lines. MMP11 gene expression in C-PBMC before and after co-culture with MCF-7 and MDA-MB-231 breast cancer cell lines (*n* = 6) (**D**,**E**) and in BC-PBMC after the same co-culture conditions (*n* = 4) (**F**,**G**). (n.s.: not significant). Results of the qRT-PCR data were represented as ΔΔCT values.

MMP1 and MMP11 do not appear to be upregulated in PBMC; however, our previous results demonstrate that MMPs are highly expressed by tumor infiltrated cells and also by surrounding sentinel lymph nodes cells in aggressive BC [11,18]. These made us hypothesize that breast cancer and/or stromal cells may have an impact on MMPs’ expression in PBMC. To test this, firstly we investigated the influence of breast cancer cells on MMP1 and MMP11 gene expression by performing PBMC-breast cancer cells co-cultures (not selected on the basis of MMP11 expression) (Figure 2A). PBMC from patients or controls showed increased MMP1 gene expression after co-culture with MCF-7 and MDA-MB-231 breast cancer cell lines, although differences were only statistically significant for C-PBMC (Figure 2B,C). Regarding MMP11 gene expression, C-PBMC showed a significant increased expression of MMP11 after being co-cultured with both breast cancer cell lines (Figure 2D,E); however, BC-PBMC did not show significant differences (Figure 2F,G). These findings suggest that MMP1 and MMP11 can be induced in C-PBMC by breast cancer cell lines, and also suggest a different regulation of MMP1 and MMP11 expression in PBMC from controls compared to PBMC from breast cancer patients.

### 2.3. MMP1 and MMP11 Gene Expression in PBMC after Co-Culture with Normal Fibroblasts or with Cancer-Associated Fibroblasts

**Figure 3:**
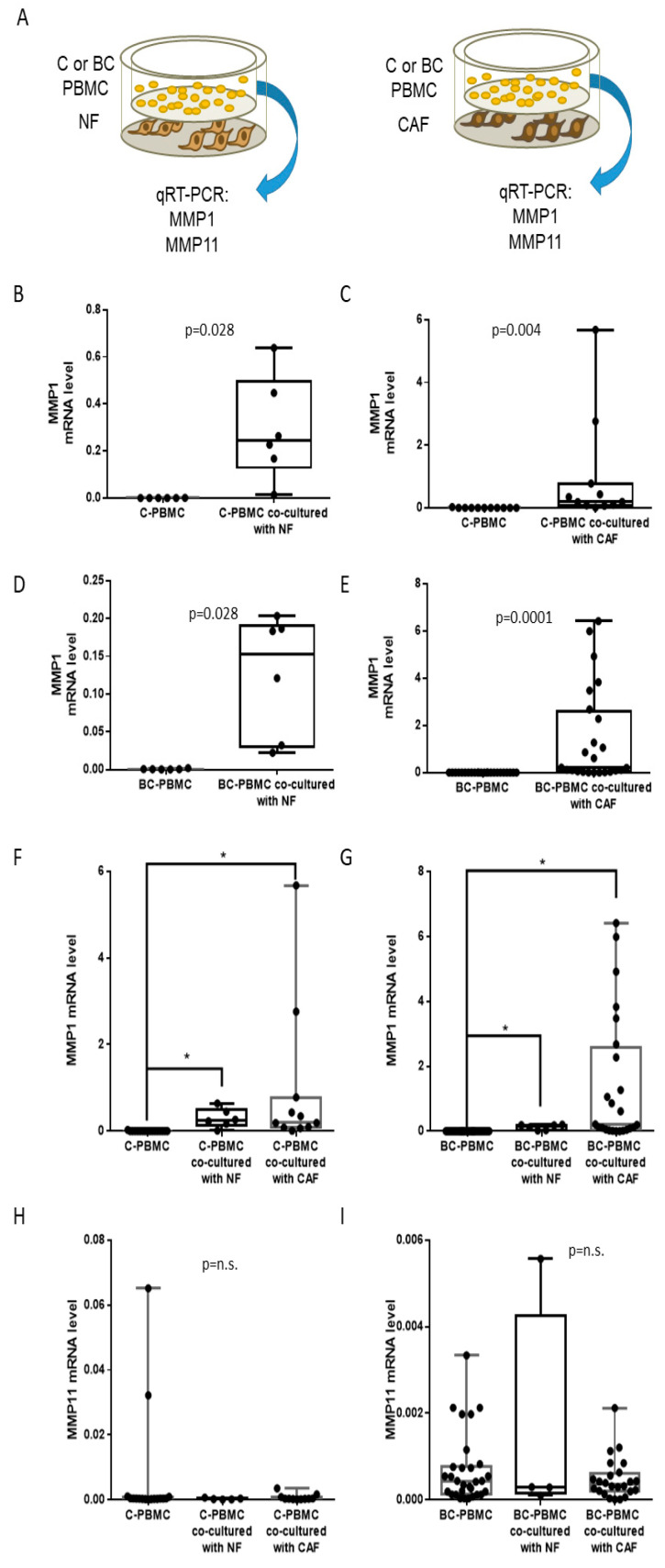
MMP1 and MMP11 gene expression in PBMC after co-culture with normal fibroblasts (NF) and cancer-associated fibroblasts (CAF). Scheme of experimental design (**A**). MMP1 gene expression in PBMC from healthy women (controls, C-PBMC) before and after co-culture with NF (*n* = 6) and CAF (*n* = 11) (**B**,**C**), and in PBMC from breast cancer patients (BC-PBMC) before and after co-culture with NF (*n* = 6) or CAF (*n* = 24) (**D**,**E**). Comparative of the MMP1 gene expression in C-PBMC and BC-PBMC after co-culture with NF (*n* = 6) and CAF (*n* = 11) (**F**,**G**). Comparative of the MMP11 gene expression in C-PBMC and BC-PBMC after co-culture with NF (*n* = 6) or CAF (*n* = 24) (**H**,**I**). Data represent the mean ± SD. (* *p* ≤ 0.05; n.s.: not significant). Results of the qRT-PCR data were represented as ΔΔCT values.

Then, we investigated the influence of tumor stromal cells on PBMC gene expression, by co-culturing PBMC (from controls and patients) together with NF or CAF (Figure 3A). Both C-PBMC (Figure 3B,C) and BC-PBMC (Figure 3D,E) showed a significant increase of MMP1 gene expression after co-culture with NF or CAF. Curiously, it was observed that MMP1 gene expression was higher when PBMC (both C-PBMC and BC-PBMC) were co-cultured with CAF rather than with NF (Figure 3F,G), although differences between co-culture with NF or CAF were not statistically significant, probably due to tumor heterogeneity. These results suggest NF and CAF can influence MMP1 gene expression in PBMC. In a different manner, MMP11 gene expression did not change significantly, either in C-PBMC or in BC-PBMC, after co-culture with NF or CAF (Figure 3H,I).

### 2.4. Influence of PBMC on the Inflammatory Profile of Breast Cancer Cell Lines, Cancer-Associated Fibroblasts and Normal Fibroblasts

**Figure 4:**
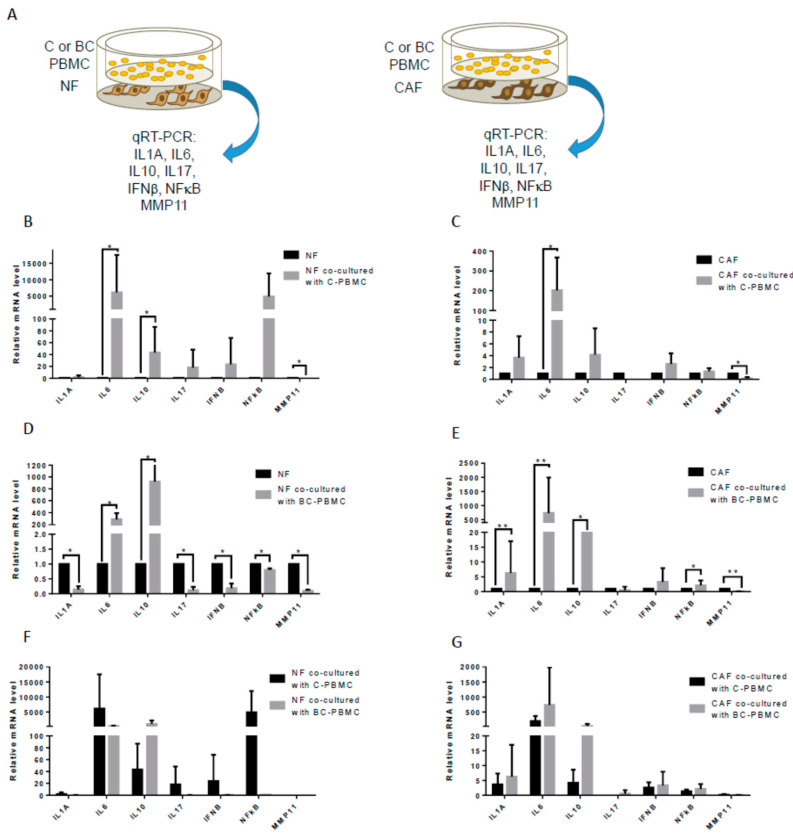
Inflammatory profile of breast cancer cell lines and fibroblasts after co-culture with PBMC. Scheme of experimental design (**A**). Gene expression of IL1A, IL6, IL10, IL17, IFNβ, NFĸB, and MMP11 in NF (*n* = 6) (**B**) and CAF (*n* = 6) (**C**) after co-culture with PBMC from healthy women (controls, C-PBMC). Gene expression of IL1A, IL6, IL10, IL17, IFNβ, NFĸB, and MMP11 in NF (*n* = 6) (**D**) and CAF (*n* = 24) (**E**) after co-culture with PBMC from breast cancer patients. Comparative of IL1A, IL6, IL10, IL17, IFNβ, NFĸB, and MMP11 gene expression in NF (**F**) and CAF (**G**) after co-culture with C-PBMC or BC-PBMC. Data represent the mean ± SD. (* *p* ≤ 0.05; ** *p* ≤ 0.01). Results of the qRT-PCR data were represented as fold expression.

We turned our attention to the influence that PBMC may exert on cancer cells. We selected a set of five inflammatory factors (IL1A, IL6, IL17A, IFNβ, and NFĸB) that were found to be overexpressed in aggressive breast carcinomas [12]. Then, we co-cultured BC-PBMC with MCF-7 and MDA-MB-231 breast cancer cell lines and we analyzed the expression of the selected genes in those breast cancer cell lines; no significant differences in their inflammatory profile were found (not shown). Then, we hypothesized that PBMC may have an impact on the gene expression of stromal cells. To test that, we performed co-cultures of C- and BC-PBMC together with NF or CAF and we measured the expression of the selected genes and also IL10 and MMP11 in fibroblasts (Figure 4A). Co-cultures of C-PBMC with either NF (Figure 4B) or CAF (Figure 4C) showed a significant increase in IL6 levels and a significant decrease in MMP11 levels, but IL10 significantly increased only in NF. Remarkably, multiple and differential changes were found when BC-PBMC were co-cultured with NF or CAF (Figure 4D,E). Co-culture of BC-PBMC with NF showed an increase of IL6 and IL10 gene expression in NF, but also a decrease of the gene expression of IL1A, IL17, IFNβ, NFĸB, and MMP11 (Figure 4D). Differently, co-culture of BC-PBMC with CAF showed significant increased expression of IL6 and IL10 in CAF, as observed in all PBMC-fibroblast co-cultures, however a specific increase of IL1A and NFĸB and a decrease of MMP11 gene expression were observed in CAF after co-culture with BC-PBMC (Figure 4E). However, no significant differences exist between co-cultures of fibroblasts (NF or CAF) with C- or BC-PBMC (Figure 4F,G).

### 2.5. Influence of PBMC on the Proliferative Capacity of Breast Cancer Cell Lines

**Figure 5:**
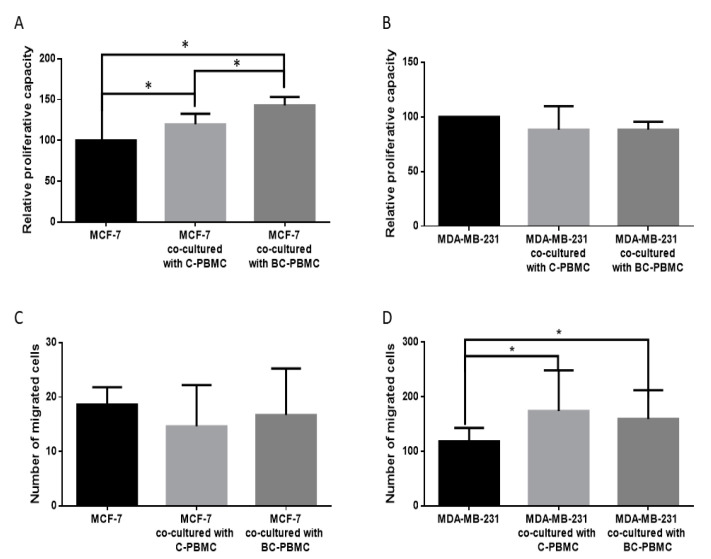
Relative proliferative capacity of breast cancer cell lines MCF-7 (**A**) and MDA-MB-231 (**B**) after cultured in presence of cm-PBMC, both from C-PBMC (*n* = 9) and BC-PBMC (*n* = 6). Invasive capacity of breast cancer cell lines MCF-7 (**C**) and MDA-MB-231 (**D**) after co-culture with C-PBMC and BC-PBMC in Matrigel invasion chambers. Data represent the mean ± SD. (* *p* ≤ 0.05).

The effect of PBMC on the proliferative capacity of breast cancer cell lines has been determined using a test based on the cleavage of tetrazolium salts added to the culture medium (WST-1, Roche, 05015944001). The tetrazolium salts are cleaved to formazan by cellular enzymes. An expansion in the number of viable cells results in an increase in the overall activity of mitochondrial dehydrogenases in the sample, which in turn increases the amount of formazan dye formed. An increased proliferation of MCF-7 has been observed when cells were cultured in presence of conditioned medium from PBMC (cm-PBMC), both from C-PBMC (*p* = 0.002) and BC-PBMC (*p* < 0.001). The increase of MCF-7 proliferation was significantly greater with BC-PBMC than with C-PBMC (*p* = 0.003) (Figure 5A). No differences have been found regarding the proliferative capacity of MDA-MB-231 (Figure 5B).

### 2.6. Influence of PBMC on the Invasiveness of Breast Cancer Cell Lines

To determine the influence of PBMC on the invasiveness of breast cancer cell lines, cell invasion assays were performed in BD BioCoatMatrigel invasion chambers. Due to the low invasiveness of MCF-7, usually employed as negative control for invasive studies, no differences in invasion capacity were observed (Figure 5C). Regarding the influence on MDA-MB-231 invasiveness, C-PBMC and BC-PBMC induced an increased invasion capacity (*p* = 0.001 and *p* = 0.002, respectively) (Figure 5D).

## 3. Discussion

Conventional mammography has a sensitivity of 66% and a specificity of 92% [19], but recent studies showed that mammography does not reduce breast cancer mortality and may lead to overdiagnosis, increasing unnecessary surgical procedures and patient anxiety [20,21]. In the last few decades, the use of serum tumor markers has been introduced for cancer screening; however, none have been proved suitable for screening the entire target population due to low specificity and sensitivity in the early stage of disease [22,23,24]. Circulating tumor cells (CTCs) detection and enumeration in breast cancer is a promising new diagnostic field, but these CTCs are present only at a ratio of 1 cell per 10^6^–10^7^ peripheral blood cells [25], which makes their detection very difficult. In contrast, PBMC are easily obtained. Then, the present data, although preliminary, may contribute to the novel concept for breast cancer detection based on the immune system response to the presence of the tumor in the body, rather than on the observation of tumor cells themselves. The present study is based on gene expression data, which should be validated at protein level and in another cohort of patients. Despite these limitations, the study provides an overview on PBMC, fibroblasts, and cancer cells interactions.

In the present study, we have investigated MMP1 and MMP11 gene expression in PBMC because of their relationship with lymph node metastasis [18] and hematogenous metastasis [6,7,8,9,10,11] in breast cancer, respectively. The analysis of the MMP11 expression in PBMC suggests the existence of a breast cancer patient subpopulation (25.9% of total patients) showing high levels of MMP11 (2-fold or higher) compared to healthy women. This fact could be of great clinical interest, although we found no significant relationship between high levels of MMP11 gene expression in BC-PBMC with clinico-pathological characteristics (data not shown). Differences in PBMC gene expression between breast cancer patients and healthy women may be related to malignancy-induced biological effects. Indeed, PBMC from breast cancer patients have previously been in contact with tumor environment. In this sense, changes regarding immune cell populations have been reported, not only in breast cancer [26,27,28], but also in other solid tumors [26,27,28,29]. In addition, according to our previous and current data, MMP11 expression is independent of tumor characteristics, such as the tumor stage, indicating that MMP11 expression might be associated with the individual’s response to the tumor, and its expression may correspond to the evolutionary stages of the tumor, at the initial stages of tumor development. This finding indicates that PBMC from some breast cancer patients are biologically different to PBMC from healthy subjects.

In order to explore the crosstalk between PBMC and tumor cells or tumor microenvironment, co-cultures of PBMC together with breast cancer cell lines or fibroblasts were performed (summary of the results in Table 1 and Table 2). MMP1 gene expression in PBMC from controls was significantly increased after co-cultures with breast cancer cell lines (MCF-7 and MDA-MB-231), and, in turn, PBMC induced an increased proliferative capacity of the mildly aggressive breast cancer cell line MCF-7, especially PBMC from breast cancer patients, and a greater invasive capacity of the highly invasive MDA-MB-231 cell line. Additionally, MMP1 gene expression in PBMC from controls was significantly increased after co-cultures with CAF or normal fibroblasts. However, MMP1 gene expression in PBMC from breast cancer patients was significantly increased only after co-cultures with CAF or normal fibroblasts, but not after co-culture with breast cancer cell lines. However, and importantly, PBMC from both controls and breast cancer patients showed a higher MMP1 gene expression after co-culture with CAF than with normal fibroblasts, suggesting an impact on MMP1 regulation from tumor microenvironment. In accordance with that, previous data from our group indicate that MMP1 overexpression in tumor-infiltrating immune cells is an early event at the microinvasive focus of in situ breast carcinomas [30]. MMP1 expression was also significantly increased in aggressive breast tumors and correlates with both tumor size and histological grade [31]. Likewise, MMP1 expression in immune cells surrounding cancer cells in positive sentinel nodes was also strongly associated with tumor involvement of non-sentinel lymph nodes in patients with invasive breast cancer [18]. In addition to these, an association between circulating tumor cells with epithelial-mesenchymal transition (CTC-EMT) and MMP1 expression in primary tumor tissue has been reported, suggesting that therapeutic targeting of MMP1 could lead to a decrease in MMP1-induced EMT and, subsequently, decrease CTC-EMT and then cause a reduction in tumor dissemination or treatment resistance [32]. Regarding MMP11 gene expression, PBMC from controls after co-culture with both breast cancer cell lines showed a significant increased expression; however, PBMC from breast cancer patients did not show significant differences. This result suggests a possible modulation of MMP11 gene expression in PBMC during an early phase of the interaction with tumor cells and the possible existence of a prior molecular interaction memory.

A key aspect in breast cancer is the role of inflammatory cells in the tumor-stroma crosstalk. In this sense, it is known that CAF contribute to tumor progression by several mechanisms, including the evocation of an inflammatory response, and, also, that CAF show a different phenotype to normal breast-associated fibroblasts [16,17,33,34]. In order to explore this scenario, gene expression of a set of seven factors, overexpressed in biologically aggressive breast carcinomas [12], were analyzed in breast cancer cell lines and CAF before and after co-culture with PBMC from breast cancer patients. No significant differences were found in breast cancer cell lines (not shown). However, IL1A, IL6, IL10, and NFĸB gene expression in CAF was increased, whereas MMP11 was decreased after co-culture with PBMC from breast cancer patients. By contrast, gene expression of IL1A, IL17, IFNβ, NFĸB, and MMP11 in normal fibroblasts was downregulated after same culture conditions. All of these data seem to indicate a special reactivity of CAF when interacting with breast cancer patients’ PBMC, which can help to better understand the context of the relationship between the immune system and tumor stroma in breast cancer. Indeed, IL10 has immunosuppressive properties and was shown to be critical in the generation of antigen-presenting cells (APCs) with tolerogenic properties [35,36] and in the prevention of self-tissue damage [37,38,39]. It has been shown that IL10 gene can be deacetylated by HDAC11 and disruption of this deacetylation induced IL10 expression and as a consequence compromises antigen-specific T-cell immune responses [40]. The fact that IL10 expression was strongly induced in NF and CAF co-cultured with BC-PBMC indicates that BC-PBMC could be instrumental in the induction of immune tolerance against breast cancer cells and thus a suppression of an anti-tumoral immune response by upregulating IL10. In addition, these results support the implication of MMP11 expression by inflammatory cells, but not by fibroblasts, in the context of dynamic tumor-stroma interactions, and its relationship with clinical outcome [15]. In addition, these inflammatory factors, besides their central role in the inflammation process, have been related to distant metastasis promotion [12] due to their role in tumor progression through several pathways, including the generation of free radicals that can induce DNA damage and mutations that can lead to tumor initiation, stimulating cell proliferation and reducing apoptosis, promoting EMT, and angiogenesis [41,42,43].

## 4. Materials and Methods

### 4.1. Patients and Controls

In this non-randomized prospective study, 54 women with a confirmed diagnosis of invasive breast carcinoma were included. Specifically, we selected consecutive T1 or T2 invasive ductal carcinoma cases, yielding enough material for cell culture and those from which a blood sample could be obtained, during the period July 2014 to August 2016. All patients included underwent tumor resection as first therapeutic approach. The exclusion criteria were: metastatic disease at presentation, prior history of a malignant tumor, bilateral breast cancer at presentation, and having received any type of therapy prior to surgery. The clinical and pathological features of the 54 patients included in this study are listed in Table 3. Additionally, blood was collected from 28 healthy women with no tumor, infection infectious pathology or immunological pathology as controls.

### 4.2. Blood Collection

PBMC were isolated by Ficoll-Hypaque density gradient centrifugation. For each participant (patients and controls), 30 mL of peripheral blood was collected into a tube containing ethylenediaminetetraacetic acid (EDTA). The samples were stored at room temperature no more than 24 h (usually 3–4 h) until they were processed and diluted 1:1 in an equal volume of phosphate buffered saline (PBS). Diluted blood was carefully added on the top of Ficoll-Hypaque (15 mL for each 30 mL diluted blood) in two 50 mL centrifuge tubes. After centrifugation at 400× *g* for 30 min (no brake), PBMC were collected from the interphase layer, placed into another centrifuge tube, and washed with PBS. After counting, PBMC were cryopreserved in complete medium containing 10% DMSO and stored at −80 °C until use.

### 4.3. Primary Cells, Breast Cancer Cell Lines and Co-Culture Assays

For the isolation of normal fibroblasts (NF) from breast reduction mammoplasties and cancer-associated fibroblasts (CAF) from breast tumors, samples were cut into 1 mm^3^ pieces and enzymatically dissociated, as previously reported [17]. MCF-7 cells were obtained from the American Type Culture Collection (ATCC, Rockville, MD, USA) before 2011, and MDA-MB-231 cells were obtained from the European Collection of Authenticated Cell Cultures (ECACC, 92020424) in 2018. These cell lines were not further authenticated. Cells were maintained in culture fewer than 20 passages. Cell lines were checked for the absence of *mycoplasma* by PCR reaction and were not contaminated by *mycoplasma* before and after experiments. The estrogen-dependent human breast cancer-derived cell line MCF-7 and the estrogen-independent human breast cancer-derived cell line MDA-MB-231 were cultured in DMEM-F12 (Lonza, Visp, Switzerland) supplemented with 10% Fetal Bovine Serum (Biowest, Nuaillé, France) and 1% penicillin-streptomycin solution (Gibco, Paisley, UK).

MCF-7 and MDA-MB-231 cell lines were co-cultured with PBMC from breast cancer patients and healthy women (controls). CAF isolated from breast tumors were co-cultured with PBMC from breast cancer patients and controls, while NF isolated from mammoplasties were co-cultured with PBMC from breast cancer patients and controls.

Cells were seeded at the bottom of 6-well cell culture plates (MCF-7: 2 × 10^5^; MDA-MB-231: 1.5 × 10^5^; CAF and NF: 1.5 × 10^5^ cells/well), whereas PBMC (6 × 10^6^) were seeded in 0.4 μm pore size tissue culture inserts. The cells were co-cultured for 48 h in DMEM-F12 and recollected for further studies.

### 4.4. qRT-PCR

The RNeasy Mini Kit (Qiagen, Hilden, Germany) was used for total RNA extraction following the manufacturer’s instructions. For cDNA synthesis, a Transcriptor First Strand cDNA Synthesis Kit (Roche, Mannheim, Germany) was used as previously reported [17]. Quantitative real time-PCR (qRT-PCR) was performed using LightCycler^®^ 480 Probes Master and RealTime ready Custom Single Assays (Roche, Mannheim, Germany) and primers (listed in Table 4). The expression was quantified using advanced relative quantification using the LightCycler software. In order to minimize sample variability and to increase the accuracy and resolution of gene expression normalization, the combination of two reference genes (β-actin and GAPDH) was used.

### 4.5. Proliferation Assay

To determine the effect of PBMC on the proliferation capacity of breast cancer cell lines, PBMC were cultured for 48 h in DMEM-F12 (2 × 10^6^ cells/mL). After 48 h, PBMC were centrifuged for 5 min at 400× *g* and the supernatant or conditioned medium of PBMC (cm-PBMC) was collected. Breast cancer cell lines were seeded in 96-well plates (MCF-7: 5 × 10^3^ cells/well, MDA-MB-231: 3.5 × 10^3^ cells/well) in DMEM-F12 supplemented with 10% Fetal Bovine Serum and 1% penicillin-streptomycin. After 24 h, the media were removed and cells were treated with 100 µL per well of cm-PBMC, using DMEM-F12 medium as a control, and cultured for 48 h. Finally, 10 µL of the WST-1 proliferation reagent (Roche, 05015944001) was added and incubated for 4 h at 37 °C and 5% CO_2_. Proliferation was quantified measuring the absorbance at 450 nm and subtracting the absorbance value at 655 nm.

### 4.6. Cell Invasion Assay

To determine the effect of PBMC on the invasiveness of breast cancer cell lines (MCF-7 and MDA-MB-231), cell invasion assays were performed in BD BioCoatMatrigel invasion chambers (24-well plates, Corning, Biocoat Matrigel™, 354480). Briefly, the breast cancer cell lines (MCF-7: 5 × 10^4^ cells; MDA-MB-231: 1 × 10^4^ cells) were seeded in DMEM-F12 in the upper chamber, whereas PBMC were seeded at 2 × 10^6^ cells/mL in DMEM-F12 in the lower chamber. DMEM-F12 without PBMC was used as control. After incubation for 48 h, cells that migrated to the lower surfaces of the filters were fixed in cold absolute methanol for 20 min, stained using a 0.5% crystal violet solution and 20% methanol for 30 min in the dark, visualized, and counted. Values for cell invasion were expressed as the mean number of cells per microscopic field over ten fields.

### 4.7. Statistical Analysis

All statistical analyses were performed using SPSS 18. The Kolmogorov-Smirnov test was used to determine whether the sample data were normally distributed. For qPCR analysis, comparisons between groups were performed using Wilcoxon test for paired samples or Mann-Whitney *U* test for median comparison. Differences were considered significant when *p* ≤ 0.05.

### 4.8. Ethics Approval and Consent to Participate

Women were treated according to the guidelines used in our institution (Fundación Hospital de Jove). Written informed consent was obtained from all patients and controls. The study adhered to national regulations and was approved by Ethical Committee of Regional Clinical Research of the Principality of Asturias (ref.: 80/2013). Informed consent was obtained from all individual participants included in the study.

## 5. Conclusions

In summary, our data provide a new angle of the molecular profile of PBMC from breast cancer patients as well as their interaction with cancer cells and stromal cells, which has an impact on the inflammatory environment of breast carcinomas. On the basis of these new concepts, further studies may improve several aspects of clinical breast cancer management, such as diagnosis, prognosis, or new therapeutic targets. 

## Figures and Tables

**Table 1:** Summary of the MMP1 and MMP11 gene expression in PBMC after co-culture.

	C-PBMC ^a^	BC-PBMC ^b^
MCF-7	↑MMP1 ↑MMP11	No significant changes
MDA-MB-231	↑MMP1 ↑MMP11	No significant changes
NF ^c^	↑MMP1	↑MMP1
CAF ^d^	↑↑MMP1	↑↑MMP1

^a^: C-PBMC: peripheral blood mononuclear cells from healthy women; ^b^: BC-PBMC: peripheral blood mononuclear cells from breast cancer patients; ^c^: NF: normal fibroblasts; ^d^: CAF: cancer-associated fibroblasts.

**Table 2:** Summary of the inflammatory selected set gene expression in fibroblast after co-culture.

	NF ^a^	CAF ^b^
C-PBMC ^c^	↑IL6 ↑IL10 ↓MMP11	↑IL6 ↓MMP11
BC-PBMC ^d^	↑IL6 ↓IL1A ↑IL10 ↓IL17 ↓IFNβ ↓NFκB ↓MMP11	↑IL6 ↑IL1A ↑IL10 ↑NFκB ↓MMP11

^a^: NF: normal fibroblasts; ^b^: CAF: cancer-associated fibroblasts; ^c^: C-PBMC: peripheral blood mononuclear cells from healthy women; ^d^: BC-PBMC: peripheral blood mononuclear cells from breast cancer patients.

**Table 3:** Basal characteristics of the 54 patients with breast cancer.

Characteristics	BC ^a^ Cases (%)
Age median (years)	
≤61	27 (50.0)
>61	27 (50.0)
Tumor size	
T1	34 (63.0)
T2	20 (37.0)
Nodal status	
N−	37 (68.5)
N+	17 (31.5)
Tumor stage	
I	27 (50.0)
II	18 (33.3)
III	9 (16.7)
Histological grade	
Well differentiated	5 (9.2)
Mod differentiated	28 (51.9)
Poorly differentiated	21 (38.9)
HER2 status	
Negative	46 (85.2)
Positive	8 (14.8)
p53 status	
Negative	30 (71.43)
Positive	12 (28.57)
Estrogen receptors	
Negative	5 (9.3)
Positive	49 (90.7)
Progesterone receptors	
Negative	7 (13.0)
Positive	47 (87.0)
Molecular subtypes	
Luminal A	21 (38.9)
Luminal B	28 (51.9)
Her2	2 (3.7)
Triple-negative	3 (5.5)
Ki67 status	
Low	21 (38.9)
High	33 (61.1)

^a^: Breast Cancer.

**Table 4:** RealTime ready Custom Single Assays.

Gene Symbol	Gene Name	References (Assay ID)
MMP1	Matrix metalloprotease 1	103943
MMP11	Matrix metalloprotease 11	147004
IL1α	Interleukin 1, alpha	145628
IL6	Interleukin 6	144013
IL17A	Interleukin 17A	136839
IFNβ1	Interferon, beta-1	145797
NFκB1	Nuclear factor kappa B	141036
β-actin	Actin, beta	143636
GAPDH	Glyceraldehyde-3-phosphate dehydrogenase	102052
**Gene Symbol**	**Gene Name**	**Primer Sequences**
IL10	Interleukin 10	5′-GCGCTGTCATCGATTTCTTC-3′ 5′-TCACTCATGGCTTTGTAGATGC-3′

## Data Availability

The data presented in this study are available on request from the corresponding author. The data are not publicly available due to their containing information that could compromise the privacy of research participants.

## References

[1] de Visser K.E., Eichten A., Coussens L.M. (2006). Paradoxical roles of the immune system during cancer development. Nat. Rev. Cancer.

[2] Lin E.Y., Pollard J.W. (2004). Role of infiltrated leucocytes in tumour growth and spread. Br. J. Cancer.

[3] Coussens L.M., Werb Z. (2002). Inflammation and cancer. Nature.

[4] Daniel D., Chiu C., Giraudo E., Inoue M., Mizzen L.A., Chu N.R., Hanahan D. (2005). CD4+ T cell-mediated antigen-specific immunotherapy in a mouse model of cervical cancer. Cancer Res..

[5] Eiro N., Fernandez-Garcia B., Gonzalez L.O., Vizoso F.J. (2013). Clinical Relevance of Matrix Metalloproteases and their Inhibitors in Breast Cancer. J. Carcinog. Mutagene.

[6] Gonzalez L.O., Pidal I., Junquera S., Corte M.D., Vazquez J., Rodriguez J.C., Lamelas M.L., Merino A.M., Garcia-Muniz J.L., Vizoso F.J. (2007). Overexpression of matrix metalloproteinases and their inhibitors in mononuclear inflammatory cells in breast cancer correlates with metastasis-relapse. Br. J. Cancer.

[7] Vizoso F.J., Gonzalez L.O., Corte M.D., Rodriguez J.C., Vazquez J., Lamelas M.L., Junquera S., Merino A.M., Garcia-Muniz J.L. (2007). Study of matrix metalloproteinases and their inhibitors in breast cancer. Br. J. Cancer.

[8] Gonzalez L.O., Corte M.D., Vazquez J., Junquera S., Sanchez R., Vina A., Rodriguez J.C., Lamelas M.L., Vizoso F. (2008). Study of matrix metalloproteinases and their tissue inhibitors in ductal in situ carcinomas of the breast. Histopathology.

[9] Gonzalez L.O., Gonzalez-Reyes S., Marin L., Gonzalez L., Gonzalez J.M., Lamelas M.L., Merino A.M., Rodriguez E., Pidal I., del Casar J.M. (2010). Comparative analysis and clinical value of the expression of metalloproteases and their inhibitors by intratumour stromal mononuclear inflammatory cells and those at the invasive front of breast carcinomas. Histopathology.

[10] Eiro N., Fernandez-Garcia B., Gonzalez L.O., Vizoso F.J. (2013). Cytokines related to MMP-11 expression by inflammatory cells and breast cancer metastasis. Oncoimmunology.

[11] Eiro N., Fernandez-Garcia B., Vazquez J., Del Casar J.M., Gonzalez L.O., Vizoso F.J. (2015). A phenotype from tumor stroma based on the expression of metalloproteases and their inhibitors, associated with prognosis in breast cancer. Oncoimmunology.

[12] Eiro N., Gonzalez L., Gonzalez L.O., Fernandez-Garcia B., Lamelas M.L., Marin L., Gonzalez-Reyes S., del Casar J.M., Vizoso F.J. (2012). Relationship between the inflammatory molecular profile of breast carcinomas and distant metastasis development. PLoS ONE.

[13] Eiró N., Pidal I., Fernandez-Garcia B., Junquera S., Lamelas M.L., del Casar J.M., González L.O., López-Muñiz A., Vizoso F.J. (2012). Impact of CD68/(CD3+CD20) ratio at the invasive front of primary tumors on distant metastasis development in breast cancer. PLoS ONE.

[14] Fernandez-Garcia B., Eiro N., Miranda M.A., Cid S., Gonzalez L.O., Dominguez F., Vizoso F.J. (2016). Prognostic significance of inflammatory factors expression by stroma from breast carcinomas. Carcinogenesis.

[15] Eiro N., Cid S., Fernandez B., Fraile M., Cernea A., Sanchez R., Andicoechea A., DeAndres Galiana E.J., Gonzalez L.O., Fernandez-Muniz Z. (2019). MMP11 expression in intratumoral inflammatory cells in breast cancer. Histopathology.

[16] Eiro N., Gonzalez L., Martinez-Ordonez A., Fernandez-Garcia B., Gonzalez L.O., Cid S., Dominguez F., Perez-Fernandez R., Vizoso F.J. (2018). Cancer-associated fibroblasts affect breast cancer cell gene expression, invasion and angiogenesis. Cell Oncol. (Dordr).

[17] Gonzalez L., Eiro N., Fernandez-Garcia B., Gonzalez L.O., Dominguez F., Vizoso F.J. (2016). Gene expression profile of normal and cancer-associated fibroblasts according to intratumoral inflammatory cells phenotype from breast cancer tissue. Mol. Carcinog..

[18] Eiro N., Gonzalez L.O., Atienza S., Gonzalez-Quintana J.M., Beridze N., Fernandez-Garcia B., Perez-Fernandez R., Garcia-Caballero T., Schneider J., Vizoso F.J. (2013). Prediction of metastatic breast cancer in non-sentinel lymph nodes based on metalloprotease-1 expression by the sentinel lymph node. Eur. J. Cancer.

[19] Pisano E.D., Gatsonis C., Hendrick E., Yaffe M., Baum J.K., Acharyya S., Conant E.F., Fajardo L.L., Bassett L., D’Orsi C. (2005). Diagnostic performance of digital versus film mammography for breast-cancer screening. N. Engl. J. Med..

[20] Gotzsche P.C., Nielsen M. (2011). Screening for breast cancer with mammography. Cochrane Database Syst. Rev..

[21] Miller A.B., Wall C., Baines C.J., Sun P., To T., Narod S.A. (2014). Twenty five year follow-up for breast cancer incidence and mortality of the Canadian National Breast Screening Study: Randomised screening trial. BMJ.

[22] Duffy M.J. (2006). Serum tumor markers in breast cancer: Are they of clinical value?. Clin. Chem..

[23] Harris L., Fritsche H., Mennel R., Norton L., Ravdin P., Taube S., Somerfield M.R., Hayes D.F., Bast R.C., American Society of Clinical Oncology (2007). American Society of Clinical Oncology 2007 update of recommendations for the use of tumor markers in breast cancer. J. Clin. Oncol..

[24] Ludwig J.A., Weinstein J.N. (2005). Biomarkers in cancer staging, prognosis and treatment selection. Nat. Rev. Cancer.

[25] Ross A.A., Cooper B.W., Lazarus H.M., Mackay W., Moss T.J., Ciobanu N., Tallman M.S., Kennedy M.J., Davidson N.E., Sweet D. (1993). Detection and viability of tumor cells in peripheral blood stem cell collections from breast cancer patients using immunocytochemical and clonogenic assay techniques. Blood.

[26] Leong P.P., Mohammad R., Ibrahim N., Ithnin H., Abdullah M., Davis W.C., Seow H.F. (2006). Phenotyping of lymphocytes expressing regulatory and effector markers in infiltrating ductal carcinoma of the breast. Immunol. Lett..

[27] Liyanage U.K., Moore T.T., Joo H.G., Tanaka Y., Herrmann V., Doherty G., Drebin J.A., Strasberg S.M., Eberlein T.J., Goedegebuure P.S. (2002). Prevalence of regulatory T cells is increased in peripheral blood and tumor microenvironment of patients with pancreas or breast adenocarcinoma. J. Immunol..

[28] Whitehead R.H., Thatcher J., Teasdale C., Roberts G.P., Hughes L.E. (1976). T and B lymphocytes in breast cancer stage relationship and abrogation of T-lymphocyte depression by enzyme treatment in vitro. Lancet.

[29] Zelig U., Barlev E., Bar O., Gross I., Flomen F., Mordechai S., Kapelushnik J., Nathan I., Kashtan H., Wasserberg N. (2015). Early detection of breast cancer using total biochemical analysis of peripheral blood components: A preliminary study. BMC Cancer.

[30] Gonzalez L.O., Gonzalez-Reyes S., Junquera S., Marin L., Gonzalez L., Del Casar J.M., Gonzalez J.M., Vizoso F. (2010). Expression of metalloproteases and their inhibitors by tumor and stromal cells in ductal carcinoma in situ of the breast and their relationship with microinvasive events. J. Cancer Res. Clin. Oncol..

[31] McGowan P.M., Duffy M.J. (2008). Matrix metalloproteinase expression and outcome in patients with breast cancer: Analysis of a published database. Ann. Oncol..

[32] Cierna Z., Mego M., Janega P., Karaba M., Minarik G., Benca J., Sedlackova T., Cingelova S., Gronesova P., Manasova D. (2014). Matrix metalloproteinase 1 and circulating tumor cells in early breast cancer. BMC Cancer.

[33] Eck S.M., Cote A.L., Winkelman W.D., Brinckerhoff C.E. (2009). CXCR4 and matrix metalloproteinase-1 are elevated in breast carcinoma-associated fibroblasts and in normal mammary fibroblasts exposed to factors secreted by breast cancer cells. Mol. Cancer Res..

[34] Eiro N., Cid S., Fraile M., Cabrera J.R., Gonzalez L.O., Vizoso F.J. (2020). Analysis of the Gene Expression Profile of Stromal Pro-Tumor Factors in Cancer-Associated Fibroblasts from Luminal Breast Carcinomas. Diagnostics (Basel).

[35] Grutz G. (2005). New insights into the molecular mechanism of interleukin-10-mediated immunosuppression. J. Leukoc. Biol..

[36] Wakkach A., Fournier N., Brun V., Breittmayer J.P., Cottrez F., Groux H. (2003). Characterization of dendritic cells that induce tolerance and T regulatory 1 cell differentiation in vivo. Immunity.

[37] Li M.O., Flavell R.A. (2008). Contextual regulation of inflammation: A duet by transforming growth factor-beta and interleukin-10. Immunity.

[38] Murai M., Turovskaya O., Kim G., Madan R., Karp C.L., Cheroutre H., Kronenberg M. (2009). Interleukin 10 acts on regulatory T cells to maintain expression of the transcription factor Foxp3 and suppressive function in mice with colitis. Nat. Immunol..

[39] Rubtsov Y.P., Rasmussen J.P., Chi E.Y., Fontenot J., Castelli L., Ye X., Treuting P., Siewe L., Roers A., Henderson W.R. (2008). Regulatory T cell-derived interleukin-10 limits inflammation at environmental interfaces. Immunity.

[40] Villagra A., Cheng F., Wang H.W., Suarez I., Glozak M., Maurin M., Nguyen D., Wright K.L., Atadja P.W., Bhalla K. (2009). The histone deacetylase HDAC11 regulates the expression of interleukin 10 and immune tolerance. Nat. Immunol..

[41] Eiro N., Vizoso F.J. (2012). Inflammation and cancer. World J. Gastrointest. Surg..

[42] Diakos C.I., Charles K.A., McMillan D.C., Clarke S.J. (2014). Cancer-related inflammation and treatment effectiveness. Lancet Oncol..

[43] Landskron G., De la Fuente M., Thuwajit P., Thuwajit C., Hermoso M.A. (2014). Chronic inflammation and cytokines in the tumor microenvironment. J. Immunol. Res..

